# The RNA Methyltransferase Complex of WTAP, METTL3, and METTL14 Regulates Mitotic Clonal Expansion in Adipogenesis

**DOI:** 10.1128/MCB.00116-18

**Published:** 2018-07-30

**Authors:** Masatoshi Kobayashi, Mitsuru Ohsugi, Takayoshi Sasako, Motoharu Awazawa, Toshihiro Umehara, Aya Iwane, Naoki Kobayashi, Yukiko Okazaki, Naoto Kubota, Ryo Suzuki, Hironori Waki, Keiko Horiuchi, Takao Hamakubo, Tatsuhiko Kodama, Seiichiro Aoe, Kazuyuki Tobe, Takashi Kadowaki, Kohjiro Ueki

**Affiliations:** a Department of Diabetes and Metabolic Diseases, Graduate School of Medicine, University of Tokyo, Tokyo, Japan; b Department of Molecular Sciences on Diabetes, Graduate School of Medicine, University of Tokyo, Tokyo, Japan; c Translational Systems Biology and Medicine Initiative (TSBMI), University of Tokyo, Tokyo, Japan; d Research Center for Advanced Science and Technology, University of Tokyo, Tokyo, Japan; e Department of Home Economics, Otsuma Women's University, Tokyo, Japan; f First Department of Internal Medicine, University of Toyama, Toyama, Japan; g Department of Molecular Diabetic Medicine, Research Institute, National Center for Global Health and Medicine, Tokyo, Japan

**Keywords:** adipocyte, diabetes, obesity

## Abstract

Adipocyte differentiation is regulated by various mechanisms, of which mitotic clonal expansion (MCE) is a key step. Although this process is known to be regulated by cell cycle modulators, the precise mechanism remains unclear. *N*^6^-Methyladenosine (m^6^A) posttranscriptional RNA modification, whose methylation and demethylation are performed by respective enzyme molecules, has recently been suggested to be involved in the regulation of adipogenesis. Here, we show that an RNA *N*^6^-adenosine methyltransferase complex consisting of Wilms' tumor 1-associating protein (WTAP), methyltransferase like 3 (METTL3), and METTL14 positively controls adipogenesis by promoting cell cycle transition in MCE during adipogenesis. WTAP, coupled with METTL3 and METTL14, is increased and distributed in nucleus by the induction of adipogenesis dependently on RNA *in vitro*. Knockdown of each of these three proteins leads to cell cycle arrest and impaired adipogenesis associated with suppression of cyclin A2 upregulation during MCE, whose knockdown also impairs adipogenesis. Consistent with this, *Wtap* heterozygous knockout mice are protected from diet-induced obesity with smaller size and number of adipocytes, leading to improved insulin sensitivity. These data provide a mechanism for adipogenesis through the WTAP-METTL3-METTL14 complex and a potential strategy for treatment of obesity and associated disorders.

## Abstract (precis)

Adipocyte differentiation is regulated by various mechanisms, of which mitotic clonal expansion (MCE) is a key step. Although this process is known to be regulated by cell cycle modulators, the precise mechanism remains unclear.

## INTRODUCTION

Obesity is a threatening issue worldwide and is associated with many diseases, including type 2 diabetes (T2D), cardiovascular disease, and cancers. In obesity, enlarged adipocytes trigger various instances of immune cell recruitment into adipose tissue, which increases proinflammatory cytokine levels, resulting in systemic insulin resistance (1–5) and atherosclerotic diseases (6). Thus, the manipulation of adipocyte differentiation and maturation could be a promising strategy for the treatment of obesity-related diseases.

Until recently little has been known about posttranscriptional modification of RNA, but recent technical advances have shed light on the most frequent modification, methylation of *N*^6^-adenosine (m^6^A), which has been demonstrated to be installed by a methyltransferase complex consisting of Wilms' tumor 1-associating protein (WTAP), methyltransferase like 3 (METTL3), and METTL14 (WMM) in mammalian cells (7–10). Although the implication of m^6^A-RNA methylation has been partly explored at molecular and cellular levels, its significance *in vivo* has not been elucidated. On the other hand, the significance of m^6^A-RNA methylation in adipogenesis has recently been reported, as fat mass and obesity-associated protein (FTO), which was previously identified by human genome-wide association studies (GWAS) with strong association with obesity (11), has been demonstrated to be a demethylase of m^6^A of RNA (12) and promotes adipogenesis *in vitro* and development of obesity *in vivo* (13–15). However, the role of a methyltransferase complex of WMM in adipocyte differentiation or obesity has not been reported.

WTAP was originally identified as a ubiquitously expressed nuclear protein interacting with Wilms' tumor 1 (16). Although WTAP was suggested to stabilize cyclin A2 mRNA, thereby promoting cell cycle transition in vascular endothelial cells (17), little has been known about the molecular function of WTAP. However, increasing numbers of studies have recently demonstrated that WTAP conforms in a complex in the nucleus with METTL3 and METTL14, which methylate *N*^6^-adenosines of RNA in mammalian cells, involving posttranscriptional mRNA metabolism, splicing, and cell cycle regulation (7–10).

For adipocyte differentiation, cell cycle progression has been demonstrated to be an essential step by many studies. When the cells at confluence are induced with chemical agents *in vitro*, the cells that undergo two rounds of cell cycle progression and proliferation can be differentiated into mature adipocytes; this process is called mitotic clonal expansion (MCE) (18). Indeed, several cell cycle-related molecules, such as p21^Cip1^, p27^Kip1^ (19), and Skp2 (20), are involved in adipocyte number regulation, and some of these and other molecules, such as cyclin D1 (21), cyclin D3 (22), cyclin-dependent kinase 4 (CDK4) (23), and CDK2 (24), regulate adipocyte differentiation. Of these, it has been reported that insufficiency of CDK2 leads to impaired adipocyte differentiation, and it plays an essential role in MCE preceding adipocyte differentiation *in vitro* (24). On the other hand, cyclin A, a partner of CDK2 and CDK1, is known to promote cell cycle transition (25); however, the role of cyclin A in adipocyte differentiation remains unexplored.

In the current study, we demonstrate that WTAP, in cooperated with METTL3 and METTL14, has a pivotal role in promoting cell cycle transition in the MCE of adipocyte differentiation. Consequently, reduction of WTAP in mice leads to protection from diet-induced obesity (DIO), thereby improving insulin sensitivity.

## RESULTS

### WTAP is required in adipocyte differentiation *in vitro*.

**FIG 1:**
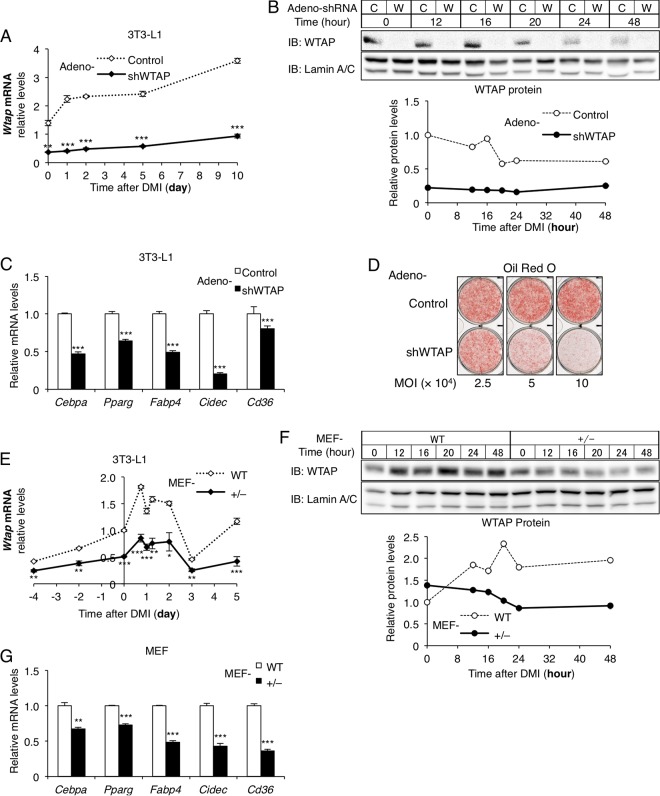
WTAP is required in adipocyte differentiation *in vitro*. (A to D) In 3T3-L1 cells WTAP was knocked down by infection of adeno-shWTAP and induced to differentiate to adipocytes (indicated as time zero). (A) mRNA expression levels of *Wtap* during 10 days after DMI (*n* = 4). (B) The protein levels of WTAP and cyclin A2 were analyzed by Western blotting and quantified with densitometry. C, adeno-control; W, adeno-shWTAP. (C) *Pparg* and its related gene expression at day 2 (*n* = 4). (D) Oil Red O staining with the indicated doses of adeno-shWTAP or control adenovirus at day 10. MOI, multiplicity of infection. (E to G) Embryonic fibroblasts from *Wtap*^+/−^ (MEF-*Wtap*^+/−^) and WT (MEF-WT) mice were induced to differentiate into adipocytes with DMI. (E) mRNA expression levels of *Wtap* before and after DMI (indicated as time zero) (*n* = 4). (F) The protein levels of WTAP were analyzed by Western blotting and quantified with densitometry. (G) *Pparg* and its related gene expression at day 2 (*n* = 4). Data represent means ± SEM (A and E) or means + SEM (C and G). *, *P* < 0.05; **, *P* < 0.01; ***, *P* < 0.001.

In order to investigate whether the WMM complex has the opposite regulatory function of *FTO* in adipocyte differentiation, we first knocked down WTAP in 3T3-L1 cells with adeno-shWTAP and induced them to differentiate into adipocytes with standard dexamethasone–3-isobutyl-1-methylxanthine (IBMX)–insulin (DMI) (Fig. 1A and B). Unexpectedly, the knockdown of WTAP suppressed adipocyte differentiation; the expression of *Pparg* and its related genes was markedly suppressed (Fig. 1C), and Oil Red O staining (Fig. 1D) also showed impaired adipocyte differentiation and maturation in a dose-dependent manner compared to that of the control adenovirus-infected cells.

Additionally, we examined the effect of WTAP reduction on adipogenesis using the embryonic fibroblasts from mice with haploinsufficiency of *Wtap* (MEF-*Wtap*^+/−^) following treatment with a standard DMI method. We observed that WTAP was constantly expressed before the induction with DMI and slightly upregulated during adipocyte differentiation at both mRNA and protein levels in embryonic fibroblasts from WT mice (MEF-WT), whereas these expression levels in MEF-*Wtap*^+/−^ were constantly maintained at lower levels (Fig. 1E and F). As a result, we found that the expression of *Pparg* and its related genes was suppressed in MEF-*Wtap*^+/−^, suggesting lower potential to differentiate into adipocytes (Fig. 1G).

### Cyclin A2 plays an essential role in adipocyte differentiation by promoting cell cycle transition during MCE.

**FIG 2:**
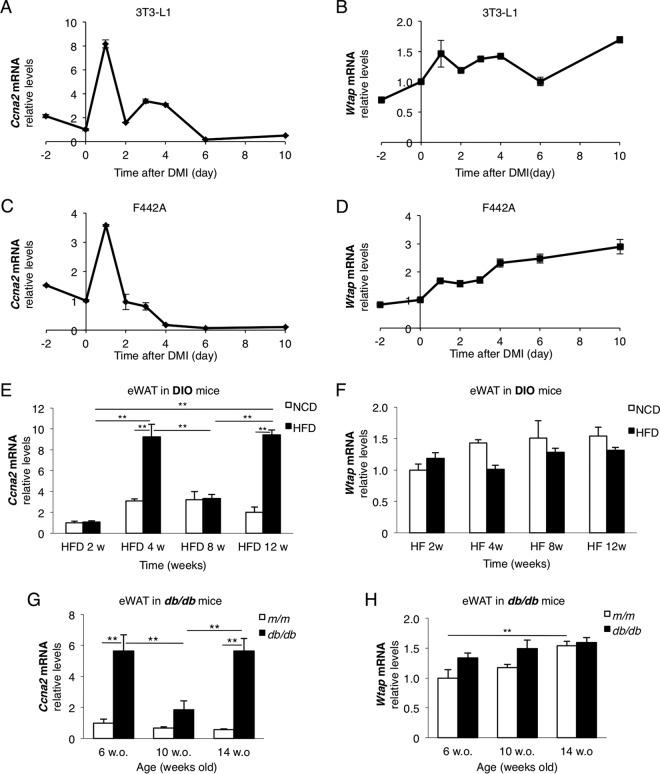
Cyclin A2 is upregulated during MCE of adipocyte differentiation *in vitro* and in the development of obesity *in vivo*. (A to D) The mRNA expressions of cyclin A2 (*Ccna2*) (A and C) and *Wtap* (B and D) in 3T3-L1 (A and B) and F442A (C and D) cells during adipocyte differentiation. Day 0 indicates the induction by standard DMI (*n* = 3). (E to H) The mRNA expressions of *Ccna2* (E and G) and *Wtap* (F and H) in the WAT of DIO mice (E and F) and *db/db* mice (G and H). C57BL/6 mice fed on HFD for the duration of the indicated weeks were compared to control mice fed on NCD (E and F), and *db/db* mice were compared to control *m/m* mice at the indicated ages (G and H) (*n* = 5). Data represent means ± SEM (A to D) or means + SEM (E to H). **, *P* < 0.01.

WTAP has been previously reported to be required for the function of cyclin A2 to promote cell cycle transition by stabilizing cyclin A2 mRNA in vascular endothelial cells (17), and cell cycle regulation has been suggested as one of the molecular functions of the WMM complex by previous ontology studies in some cell types (7–9). Moreover, the role of cyclin A2 in adipocyte differentiation remains unknown, while CDK2, a functional partner of cyclin A in promoting the cell cycle, has previously been demonstrated to play an important role during MCE of adipocyte differentiation in 3T3-L1 cells (24). Therefore, we investigated whether cyclin A2 has some roles in adipocyte differentiation through cell cycle regulation coordinately with WTAP. In 3T3-L1 cells, cyclin A2 gene (*Ccna2*) expression was transiently elevated, especially for 2 days after the induction of differentiation into adipocytes, which exactly corresponds to the MCE period (Fig. 2A), while the *Wtap* mRNA showed relatively mild upregulation during MCE and was steadily upregulated throughout adipocyte maturation (Fig. 2B). Similar upregulation of *Ccna2* and *Wtap* mRNA was observed in 3T3-F442A cells (Fig. 2C and D). We also investigated the gene expression of *Ccna2* and *Wtap* in epididymal white adipose tissue (eWAT) of DIO mice (Fig. 2E and F) and *db/db* mice (Fig. 2G and H). The eWAT of C57BL/6 mice fed on high-fat diet (HFD) exhibited transient and periodic elevations of *Ccna2* compared to that of mice fed on normal chow diet (NCD) (Fig. 2E). The WAT of *db/db* mice also exhibited similar changes in *Ccna2* mRNA compared to that of control *m/m* mice during the development of obesity (Fig. 2G). On the other hand, the upregulation of *Wtap* was less prominent than that of *Ccna2* in both *in vivo* models (Fig. 2F and H). These data suggest that cyclin A2 plays a role in adipocyte differentiation during MCE *in vitro*. In addition, *in vivo* data suggest an involvement of cyclin A2 in development of obesity.

**FIG 3:**
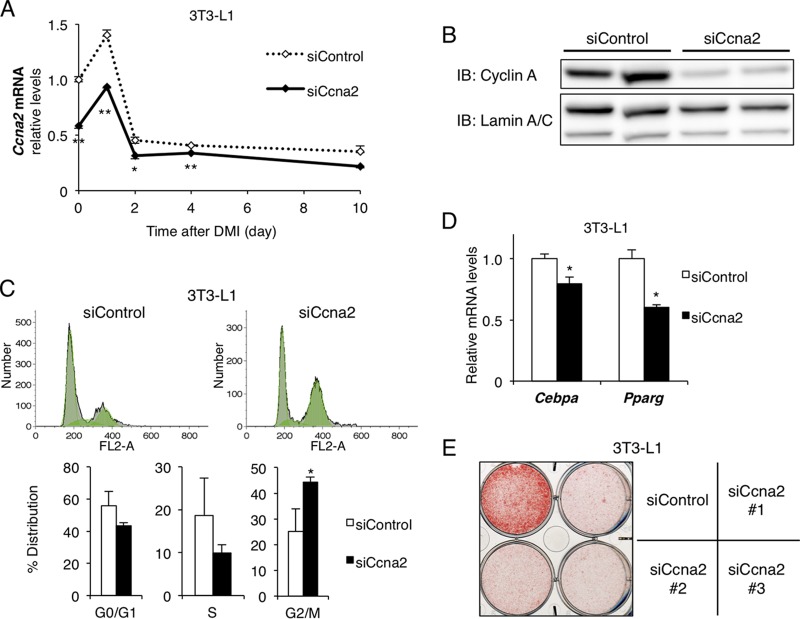
Cyclin A2 is required for cell cycle transition of MCE in adipocyte differentiation *in vitro*. Cyclin A2 was knocked down in 3T3-L1 cells with siRNA and induced to differentiate into adipocytes. (A and B) Cyclin A2 was reduced especially during the MCE period (day 0 to 2), shown at the mRNA (*n* = 3) (A) and protein (B) levels (at day 1). (C) Cell cycle analysis with flow cytometry at 40 h after standard DMI induction (*n* = 6). The typical data are shown in the upper graphs, and the lower graphs show the percentage of the distribution in each cell cycle period. (D and E) Adipocyte differentiation was evaluated by mRNA expression of *Cebpa* and *Pparg* at day 4 (*n* = 3) (D) and Oil Red O staining at day 10 (E). Data represent means ± SEM (A) or means + SEM (C and D). *, *P* < 0.05; **, *P* < 0.01.

We next knocked down cyclin A2 in 3T3-L1 cells with short interfering RNA (siRNA) and induced them to differentiate into adipocytes with standard DMI. Reductions in the levels of cyclin A2 mRNA (Fig. 3A) and protein (Fig. 3B) during MCE (days 0 to 2) resulted in cell cycle arrest, as shown by flow cytometric analysis (Fig. 3C), and impaired adipocyte differentiation, as determined by the gene expression patterns (Fig. 3D). Moreover, the Oil Red O staining also showed suppression of adipocyte differentiation in 3T3-L1 cells with three different si*Ccna2* probes (Fig. 3E).

### The knockdown of WTAP impairs cyclin A2 upregulation and arrests cell cycle transition during MCE of adipocyte differentiation *in vitro*.

**FIG 4:**
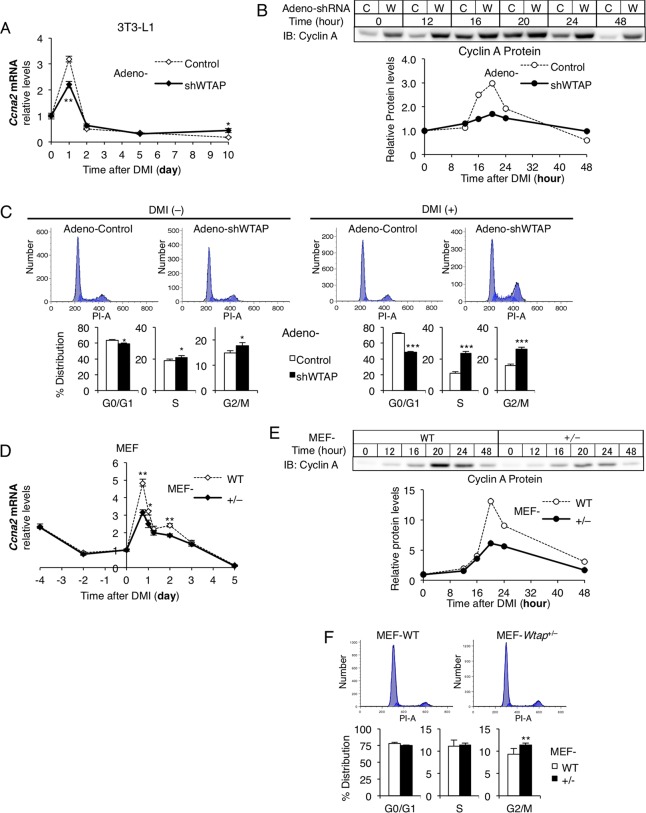
Knockdown of WTAP impairs cyclin A2 upregulation and arrests cell cycle transition during MCE of adipocyte differentiation *in vitro*. (A and B) Cyclin A2 mRNA (A) and protein (B) levels in the knockdown of WTAP with adeno-shWTAP in the same experiment as that shown in Fig. 1A and B, respectively. The protein levels of cyclin A2 were analyzed by Western blotting and quantified with densitometry. C, adeno-control; W, adeno-shWTAP. (C) Flow cytometric analysis of the cell cycle of adeno-shWTAP or control adenovirus-infected 3T3-L1 cells 40 h after DMI treatment. The typical data are shown in the upper graphs, and the lower graphs show the percentage of the distribution of each cell cycle period (*n* = 6). The left and right graphs indicate the cells without and with the induction of differentiation by DMI, respectively. (D and E) Cyclin A2 mRNA (D) and protein (E) levels in MEF-*Wtap*^+/−^ and MEF-WT mice in the same experiment as that shown in Fig. 1E and F, respectively. The protein levels of cyclin A2 were analyzed by Western blotting and quantified with densitometry. (F) Flow cytometric analysis of the cell cycle of MEF-*Wtap*^+/−^ and MEF-WT 40 h after DMI. The typical data are shown in the upper graphs, and the lower graphs show the percentage of the distribution in each cell cycle period (*n* = 4 to 6). (A, B, D, and E) Cyclin A2 mRNA and protein levels were shown as a ratio to the baseline. Data represent means ± SEM (A and D) or means + SEM (C and F). *, *P* < 0.05; **, *P* < 0.01; ***, *P* < 0.001.

The result that cyclin A2 is required in adipocyte differentiation to promote cell cycle transition in MCE prompted us to assess whether the reduction of WTAP seen in Fig. 1 affected cyclin A2. Indeed, we found that cyclin A2 upregulation relative to the basal level was impaired in 3T3-L1 cells with the WTAP knockdown compared to that of the control cells at both the mRNA (Fig. 4A) and protein (Fig. 4B) levels. In this analysis, while the absolute protein levels of cyclin A were increased overall by the WTAP knockdown, relative elevation of cyclin A protein during MCE was impaired (Fig. 4B). We also found that the cell cycle transition during MCE in adeno-shWTAP-infected cells was arrested (Fig. 4C). In this analysis, the cell cycle arrest was much more remarkable when cells were induced to differentiate with DMI rather than without DMI, indicating that WTAP and cyclin A2 have an important role in adipocyte differentiation, especially in MCE. Similarly, in MEF-*Wtap*^+/−^ the relative upregulation of cyclin A2 was also inhibited at both the mRNA (Fig. 4D) and protein (Fig. 4E) levels, and the cell cycle transition was arrested (Fig. 4F) compared to that of MEF-WT.

Taking the findings shown in Fig. 1 to 4 together, WTAP and cyclin A2 play an essential role in adipocyte differentiation by promoting cell cycle transition during MCE. Therefore, in the following analysis regarding the function of the WMM complex, we evaluated the involvement of cyclin A2 as a cell cycle regulator that is upregulated specifically in MCE, has a role close to that of WTAP, and is suppressed by WTAP reduction.

### WTAP, METTL3, and METTL14 are increased and distributed in the nucleus during adipocyte differentiation in 3T3-L1 cells.

**FIG 5:**
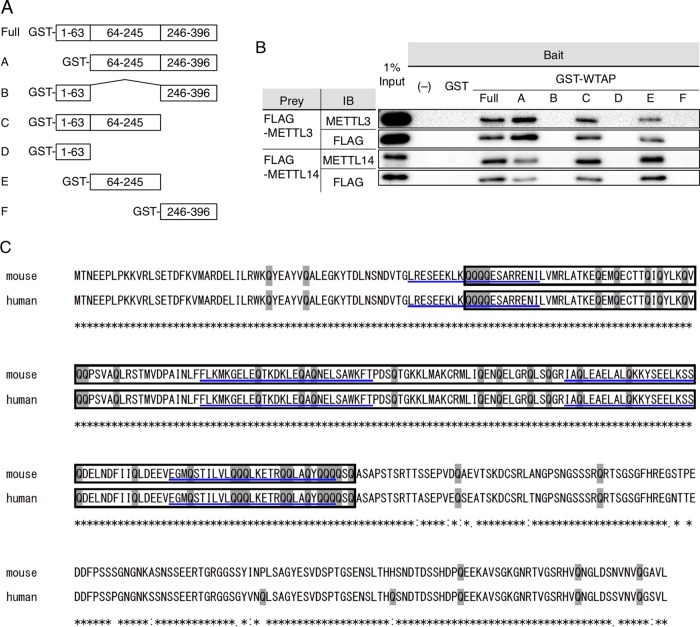
WTAP physically interacts with METTL3 and METTL14. (A and B) GST pulldown assay. (A) Schematic constructs of GST-fused full-length WTAP and its deletion mutant proteins as bait proteins. The numbers represent the amino acid numbers for the sequences of the proteins. (B) FLAG-METTL3- and FLAG-METTL14-overexpressed COS cell lysates, as prey proteins, were incubated with the immobilized bait proteins. The eluted protein complex was immunoblotted with anti-METTL3, anti-METTL14, and anti-FLAG M2 antibody. (C) The molecular structure of human and mouse WTAP based on Ensembl. The boxed sequence represents the glutamine-rich region, which corresponds to the middle region shown in Fig. 6A, with shaded glutamine residues (Q). Four coiled-coil domains are shown with underlining. A total of 96% amid acid sequence is conserved between mouse and human, with 100% conservation in the middle and N-terminal regions.

Although WTAP itself does not have any methyltransferase activity (8), it has been suggested recently that WTAP forms a nuclear protein complex with METTL3 and METTL14 and that WTAP plays a role in various biological processes, such as mRNA metabolism, splicing, and the cell cycle by recruiting METTL3 and METTL14 onto RNA in the nucleus (9). Thus, we hypothesized that WTAP regulates MCE of adipocyte differentiation through the recruitment of METTL3 and METTL14, as previously proposed (9). Indeed, the glutathione *S*-transferase (GST) pulldown assay revealed the interaction between full-length GST-WTAP and METTL3/14, and that the middle region of WTAP protein is responsible for the interaction with METTL3 and METT14 (Fig. 5A and B), which is almost consistent with a very recent study which mapped the binding surface within the WMM complex (26). This middle region corresponds to a glutamine-rich region and has four coiled-coil domains (Fig. 5C), both of which support the molecular function regarding interaction or aggregation (27, 28).

**FIG 6:**
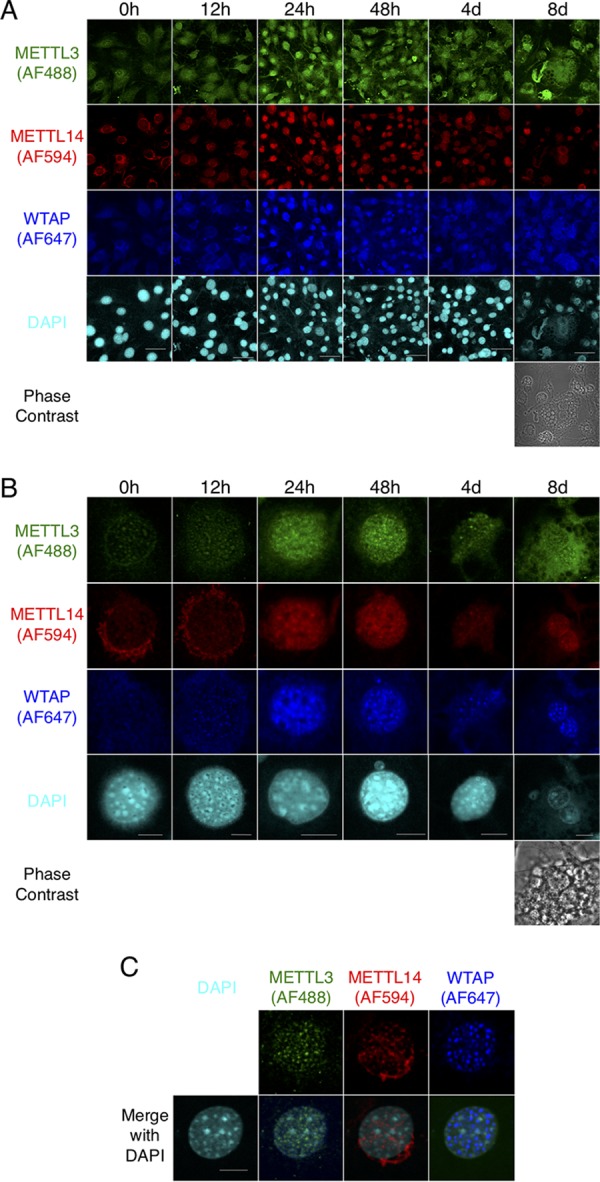
Immunofluorescence staining of WTAP, METTL3, and METTL14 in nuclei is enhanced in adipocyte differentiation of 3T3-L1 cells. Immunofluorescence study in 3T3-L1 cells, shown with METTL3-Alexa Fluor 488 (AF488) (green), METTL14-AF594 (red), WTAP-AF647 (blue), and DNA-DAPI (cyan). (A and B) The time course of METTL3, METTL14, and WTAP staining during adipocyte differentiation by DMI induction. (C) The merged images of DAPI with METTL3, METTL14, and WTAP in the cell without DMI induction. Scale bars, 50 μm (A) and 10 μm (B and C).

Interestingly, immunofluorescence staining of WTAP, METTL3, and METTL14 in nuclei was enhanced by the induction of adipocyte differentiation with standard DMI (Fig. 6A and B). These three proteins were stained mainly in nuclear speckles before the induction with DMI, not merged with 4′,6-diamidino-2-phenylindole (DAPI) (Fig. 6C), suggesting their localization in interchromatin granule clusters (IGCs), as previously reported (8, 9, 16).

**FIG 7:**
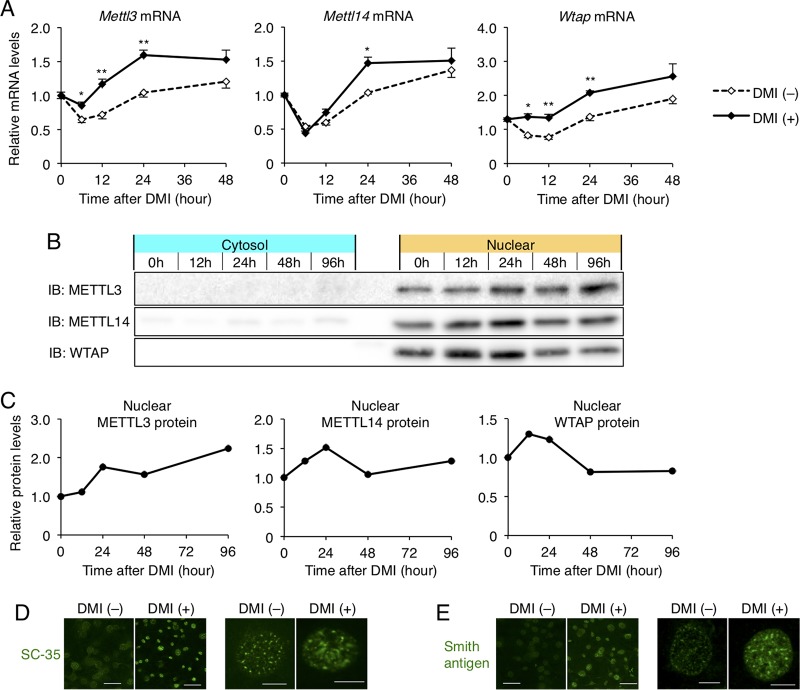
WTAP, METTL3, and METTL14 are increased and distributed in nucleus by DMI stimulation, similar to other nuclear speckle proteins, *in vitro*. (A) The mRNA expression of *Mettl3*, *Mettl14*, and *Wtap* for 48 h after DMI stimulation. Data represent means ± SEM. *P* < 0.05 (*) and *P* < 0.01 (**) for DMI (−) versus DMI (+) at each time point. (B and C) The cytoplasmic and nuclear protein levels of METTL3, METTL14, and WTAP were analyzed by Western blotting (B) and quantified by densitometry (C). (D and E) Immunofluorescence study for nuclear speckle marker protein SC-35 (D) and Smith antigen (E) in 3T3-L1 cells 24 h after DMI stimulation. Scale bars, 50 μm (left) and 10 μm (right).

Since the enhancement of immunofluorescence staining of WMM after the induction was observed not only in nuclear speckle but also in nucleoplasm (Fig. 6B) and upregulation of mRNA levels (Fig. 7A) and protein levels of WMM (Fig. 7B and C) was relatively mild, the enhancement of nuclear staining of WMM may partly represent the increased distribution of these proteins in nucleoplasm, as well as their quantitative increase. In addition, we found that immunofluorescence staining of other proteins of nuclear speckle markers, such as SC-35 (Fig. 7D) and Smith antigen (Fig. 7E), was also enhanced in both nuclear speckles and nucleoplasm by the DMI stimulation in 3T3-L1 cells.

### WTAP may recruit METTL3 and METTL14 to RNA in adipocyte differentiation *in vitro*.

**FIG 8:**
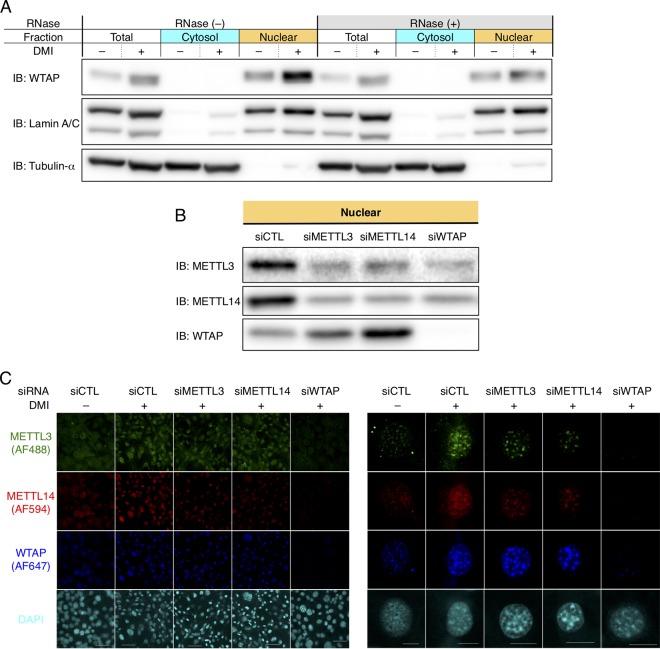
WTAP may recruit METTL3 and METTL14 to RNA in adipocyte differentiation *in vitro*. (A) 3T3-L1 cells 48 h after the induction by DMI were harvested and treated with RNase or left untreated. Their cytosolic, nuclear, and total proteins were extracted and immunoblotted with anti-WTAP antibody. Note that the increase of nuclear WTAP by DMI was canceled by RNase treatment. The fractionation of nuclear and cytosolic protein was validated by immunoblotting of lamin A/C and α-tubulin, respectively. (B and C) The effect of knockdown of METTL3, METTL14, and WTAP 24 h after DMI treatment in 3T3-L1 cells. (B) The nuclear protein levels analyzed by Western blotting. (C) The immunofluorescence study, shown with METTL3-Alexa Fluor 488 (AF488) (green), METTL14-AF594 (red), WTAP-AF647 (blue), and DNA-DAPI (cyan). Scale bars, 50 μm (left) and 10 μm (right).

To examine whether the increase of WTAP during MCE depends on RNA, we analyzed the nuclear protein with or without RNase administration during MCE of 3T3-L1 cells (Fig. 8A). The DMI-induced nuclear increase of WTAP protein was abolished after the RNase treatment, suggesting that the increase of WTAP during MCE depends on RNA. In addition, Western blotting and immunofluorescence analysis together revealed that the knockdown of WTAP reduced both METTL3 and METTL14, while the knockdown of either METTL3 or METTL14 did not reduce, and even increased, WTAP, although the single knockdown of METTL3 or METTL14 reduced each other (Fig. 8B and C). This was apparent in the Western blotting (Fig. 8B), consistent with the immunofluorescence analysis (Fig. 8C) and a previous study (9). Taken together, the nuclear increase and distribution of METTL3 and METTL14 depend on WTAP, of which the target is RNA, suggesting that WTAP has a role in recruiting METTL3 and METTL14 to RNA for activated posttranscriptional regulation in adipocyte differentiation.

### The WMM complex regulates the cell cycle transition during MCE.

**FIG 9:**
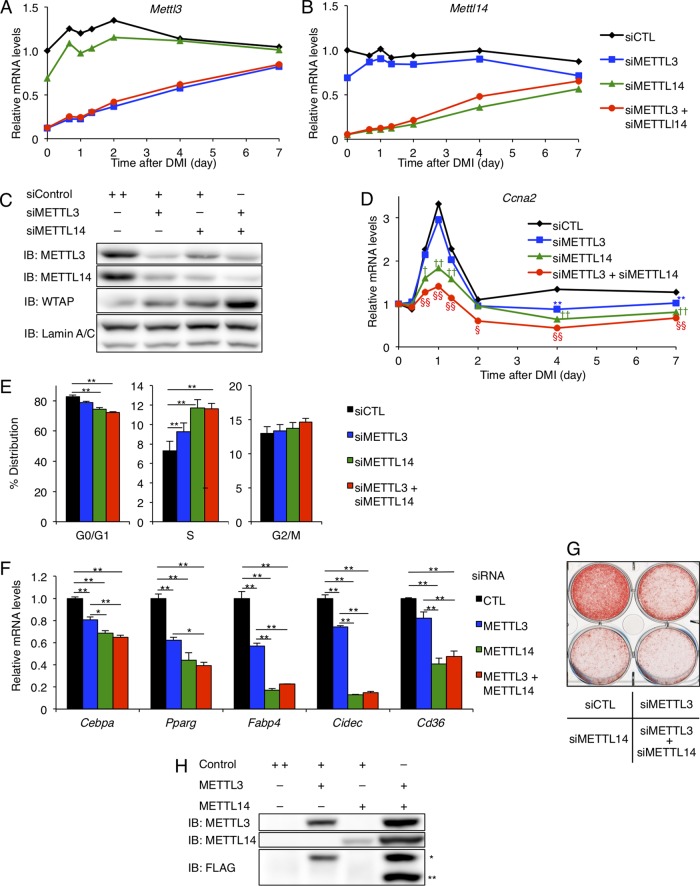
WMM complex regulates the cell cycle transition during MCE. (A to G) Single or double knockdown of METTL3 and/or METTL14 by siRNA in 3T3-L1 cells. (A and B) *Mettl3* (A) and *Mettl14* (B) mRNA expression levels after the stimulation of adipocyte differentiation with DMI (*n* = 4). (C) The protein levels of METTL3, METTL14, and WTAP 24 h after DMI treatment. (D to G) The effects of METTL3 and/or METTL14 knockdown on differentiation into adipocytes. (D) *Ccna2* mRNA expression after DMI, shown as a ratio to the baseline (*n* = 4). (E) Flow cytometric analysis of the cell cycle 40 h after DMI treatment (*n* = 5). (F) mRNA expression of *Pparg* and its related genes 48 h after DMI (*n* = 4). (G) Oil Red O staining 10 days after DMI. (H) Cooverexpression of METTL3-FLAG and METTL14-FLAG in COS cells. The empty pEZ vector was used as the control vector. In the FLAG blotting, the upper bands (*) represent FLAG-METTL3, and the lower bands (**) represent FLAG-METTL14. Data represent means (A, B, and D) or means + SEM (E and F). (D) *P* < 0.05 (*) and *P* < 0.05 (**) for siControl versus siMETTL3. *P* < 0.05 (†) and *P* < 0.01 (††) for siControl versus siMETTL14. *P* < 0.05 (§) and *P* < 0.01 (§§) for siControl versus siMETTL3 and siMETTL14. (E and F) *, *P* < 0.05; **, *P* < 0.05.

We next examined the function of METTL3 and METTL14 in adipocyte differentiation by their knockdown in 3T3-L1 cells (Fig. 9A to C). While some METTL3 and METTL14 remained at the Western blotting and immunofluorescence levels (Fig. 8B and C and 9C), these siRNAs effectively knocked down their mRNA levels during MCE (Fig. 9A and B) and exhibited the following biological effects: reductions in METTL3 and/or METTL14 impaired the relative upregulation of *Ccna2* during the MCE period (Fig. 9D) with a cell cycle arrest (Fig. 9E), with the protein level of WTAP even being upregulated (Fig. 9C). The expression of *Pparg* and its related genes (Fig. 9F) and Oil Red O staining (Fig. 9G) consistently showed inhibition of adipocyte differentiation by the knockdown of METTL3 and/or METTL14. These results suggest that METTL3 and MTTL14 are necessary for the function of the WMM complex to induce MCE in adipocyte differentiation by promoting cell cycle transition, since the effects of the knockdown of METTL3 and/or METTL14 could not be compensated for by the upregulation of WTAP.

Double knockdown of METTL3 and METTL14 showed the additive effects on adipocyte differentiation, although the effects by the METTL14 knockdown were more remarkable than those by the METTL3 knockdown (Fig. 9D to G). However, it is unclear whether METTL14 has the dominant role over METTL3 in adipocyte differentiation, since knockdown of METTL3 or METTL14 alone leads to a decrease in the other's protein level (Fig. 8B and 9C) and the cooverexpression of METTL3 and METTL14 resulted in much higher levels of the both proteins than single overexpression of either (Fig. 9H).

### *Wtap*^+/−^ mice are resistant to HFD-induced obesity, with small size and number of adipocytes.

Based on the findings regarding the role of WTAP in cultured adipocytes, to assess its role in the development of obesity, we investigated the effect of WTAP reduction *in vivo* using *Wtap*^+/−^ mice, since *Wtap*^−/−^ mice are embryonically lethal at embryonic day 6.5, causing defective formation of endoderm and ectoderm (17, 29), the etiology of which is quite similar to that of cyclin A2-null mice (30).

**FIG 10:**
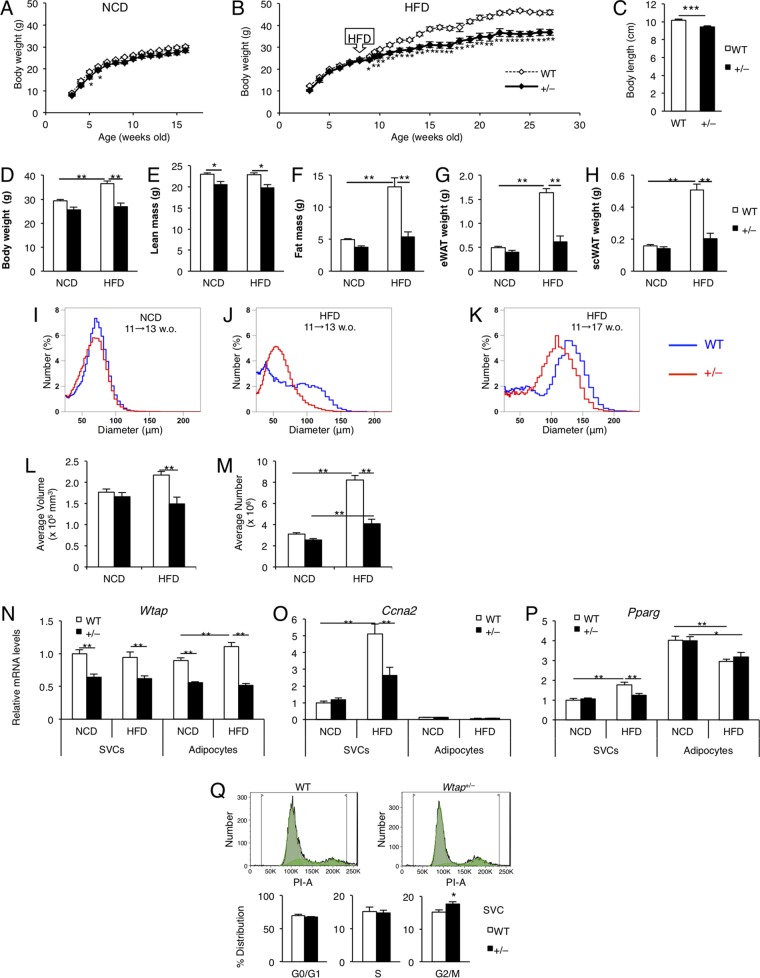
*Wtap*^+/−^ mice are resistant to HFD-induced obesity, with small size and number of adipocytes. (A and B) The time course of the change in body weight (BW) of mice fed on NCD (*n* = 7 to 14) (A) and HFD (*n* = 7 or 8) (B) (HFD during 8 to 28 weeks of age). (C) The body length of *Wtap*^+/−^ and WT mice. The mice were 16 weeks old and fed on HFD for 6 weeks (*n* = 7 to 10). (D to M) *Wtap*^+/−^ and WT mice were fed on HFD or NCD for 2 weeks (during 11 to 13 weeks of age), except for those depicted in Fig. 5K, which were fed on HFD for 6 weeks (during 11 to 17 weeks of age) and sacrificed for the following analysis. (D) Total body weight. (E and F) Lean mass (E) and fat mass (F) as measured by DEXA. (G and H) The weight of eWAT (G) and scWAT (H) (*n* = 10 or 11). (I to K) Averaged distribution of adipocyte size as measured by a Coulter counter in the eWAT from wild-type (blue) and *Wtap*^+/−^ (red) mice. (L) Average volume of one adipocyte. (M) Average number of adipocytes in the eWAT of a mouse (*n* = 7 to 11). (N to P) The gene expression of *Wtap* (N), *Ccna2* (O), and *Pparg* (P) in stromal vascular cells (SVCs) and the adipocyte fraction of eWAT from mice fed on HFD for 2 weeks at 11 to 13 weeks of age compared to those from mice fed on NCD (*n* = 8). (Q) Flow cytometric analysis of the cell cycle for the lineage and CD31 double-negative population of SVCs at the second passage (more than 96% of total SVCs) 20 h after DMI treatment. The typical data are shown in the upper graphs, and the lower graphs show the percentage of the distribution in each cell cycle period (*n* = 8 to 10). Data represent means ± SEM (A and B) or means + SEM (C to Q). *, *P* < 0.05; **, *P* < 0.01; ***, *P* < 0.001.

The body weight (BW) of *Wtap*^+/−^ mice was significantly lower than that of the wild-type (WT) mice fed on NCD; the difference became much more evident under HFD conditions (Fig. 10A, B, and D). This was mainly due to the reduced adiposity, as measured by dual-energy X-ray absorptiometry (DEXA) (Fig. 10D to F), rather than the slightly shorter body length of *Wtap*^+/−^ mice compared to that of WT mice (Fig. 10C). Indeed, the increase in the weight of eWAT (Fig. 10G) and subcutaneous WAT (scWAT) (Fig. 10H) for 2 weeks on HFD was dramatically suppressed in *Wtap*^+/−^ mice compared to that in WT mice.

To verify whether adiposity of *Wtap*^+/−^ mice is decreased by a similar mechanism observed *in vitro*, we performed the following examinations. First, we analyzed the size and number of adipocytes using a Coulter counter, and we revealed that 2 weeks on HFD increased the prevalence of both large and small adipocytes in WT mice (Fig. 10I and J), shifting to a single peak composed of large adipocytes after being on HFD for 6 weeks (Fig. 10K). Conversely, in *Wtap*^+/−^ mice, the levels of neither large nor small cells were increased to the same extent by 2 weeks on HFD (Fig. 10I and J), and the peak of the distribution shifted to cells smaller than those of WT mice after 6 weeks on HFD (Fig. 10K). Consequently, *Wtap*^+/−^ mice exhibited lower fat mass in both eWAT and scWAT because of not only the impaired expansion of the volume of individual cells (Fig. 10L) but also the smaller increase in the number of adipocytes (Fig. 10M) over the 2 weeks on HFD. The increase in the number of small cells in WT mice after 2 weeks on HFD indicates the existence of an MCE-like process *in vivo*, while the lack of an increase in small cells and resultant decrease in large cells in *Wtap*^+/−^ mice suggest the impairment of the MCE-like process *in vivo* (Fig. 10I to K).

We next fractionated adipose tissue into adipocytes and stromal vascular cells (SVCs), the latter of which include preadipocytes as well as vascular and immune cells, and analyzed the gene expression levels. *Wtap* mRNA was almost equally expressed in both SVCs and adipocytes, and it constantly decreased by half in *Wtap*^+/−^ mice compared to that in WT mice (Fig. 10N). On the other hand, *Ccna2* mRNA was almost exclusively expressed in SVCs and was highly induced by HFD for 2 weeks in the SVCs of WT mice, while that of *Wtap*^+/−^ mice was markedly suppressed (Fig. 10O). We also found that *Pparg* expression was suppressed only in SVCs from the *Wtap*^+/−^ mice fed on HFD for 2 weeks (Fig. 10P). Moreover, we evaluated the cell cycle of SVCs, which were cultured for two passages and stimulated with standard DMI. By using a flow cytometer we analyzed the cell cycle in the lineage-negative and CD31-negative population, which consisted of more than 96% used SVCs, and found that the cell cycle transition was arrested in *Wtap*^+/−^ mice compared to that in WT mice, which could also support the effect of WTAP reduction on MCE-like processes *in vivo* (Fig. 10Q).

### *Wtap*^+/−^ mice exhibited increased energy expenditure and improved leptin sensitivity and circulating lipid profiles.

**FIG 11:**
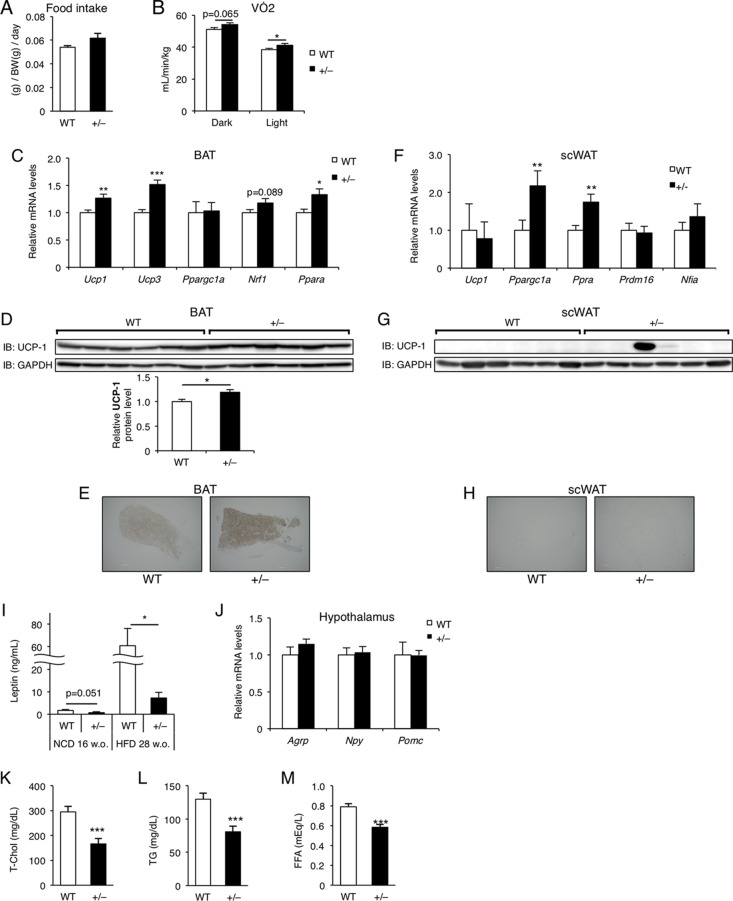
Increased energy expenditure and improved leptin sensitivity and circulating lipid profiles in *Wtap^+/^*^−^ mice. (A) Food intake at 28 weeks of age on HFD (*n* = 5). (B) O_2_ consumption measured in 7- to 9-week-old mice fed on HFD (*n* = 10). (C and F) The expression of brown fat-related genes in BAT (C) and scWAT (F) in 13-week-old mice fed on HFD for 2 weeks (*n* = 7 or 8). (D, E, G, and H) UCP-1 protein levels analyzed by Western blotting (*n* = 6) (D and G) and immunohistochemistry (E and H) in BAT (D and E) and scWAT (G and H) of 15-week-old mice fed on HFD for 2 weeks. Scale bars, 500 μm. (I) Plasma leptin levels in 16-week-old mice fed on NCD and 28-week-old mice fed on HFD for 20 weeks (*n* = 6 to 8). (J) The gene expression of neuropeptides associated with appetite in the hypothalamus in 28-week-old mice fed on HFD for 20 weeks (*n* = 8). (K to M) Plasma lipid profile from the mice fed on HFD for 20 weeks at 28 weeks of age (*n* = 6 to 15). Shown are total cholesterol (T. Chol) (K), triglycerides (TG) (L), and free fatty acids (FFA) (M). Data represent means + SEM. *, *P* < 0.05; **, *P* < 0.01; ***, *P* < 0.001.

While food intake by BW did not significantly differ between *Wtap*^+/−^ and WT mice (Fig. 11A), O_2_ consumption was increased (Fig. 11B), suggesting increased energy expenditure in *Wtap*^+/−^ mice. As one of the possible causes, *Ucp1* and its related gene expression levels were elevated in brown adipose tissue (BAT) (Fig. 11C). The increased UCP-1 protein level was also confirmed by Western blotting (Fig. 11D) and immunohistochemistry (Fig. 11E). On the other hand, the *Ucp1* mRNA levels in scWAT were not significantly altered between WT and *Wtap*^+/−^ mice (Fig. 11F). Although the gene expression levels of *Ppargc1a* and *Ppara* in scWAT of *Wtap*^+/−^ mice were significantly elevated (Fig. 11F) and UCP-1 protein was slightly positive in only a few scWAT of *Wtap*^+/−^ mice by Western blotting (Fig. 11G), the absolute UCP-1 protein levels in scWAT were far lower than those in BAT and were almost undetectable by Western blotting and immunohistochemistry in most scWAT of both WT and *Wtap*^+/−^ mice (Fig. 11G and H).

Circulating leptin levels were dramatically decreased in *Wtap*^+/−^ mice (Fig. 11I), even though the expression of neuropeptides controlling the appetite in the hypothalamus was not altered (Fig. 11J), suggesting that leptin sensitivity was elevated in *Wtap*^+/−^ mice.

As for the circulating lipid profiles, total cholesterols (T-Chol), triglycerides (TG), and free fatty acids (FFA) (Fig. 11K to M) were lower in *Wtap*^+/−^ mice than in WT mice under *ad libitum* feeding conditions.

### Hepatic steatosis and macrophage infiltration in both adipose tissue and the liver were attenuated in *Wtap*^+/−^ mice.

**FIG 12:**
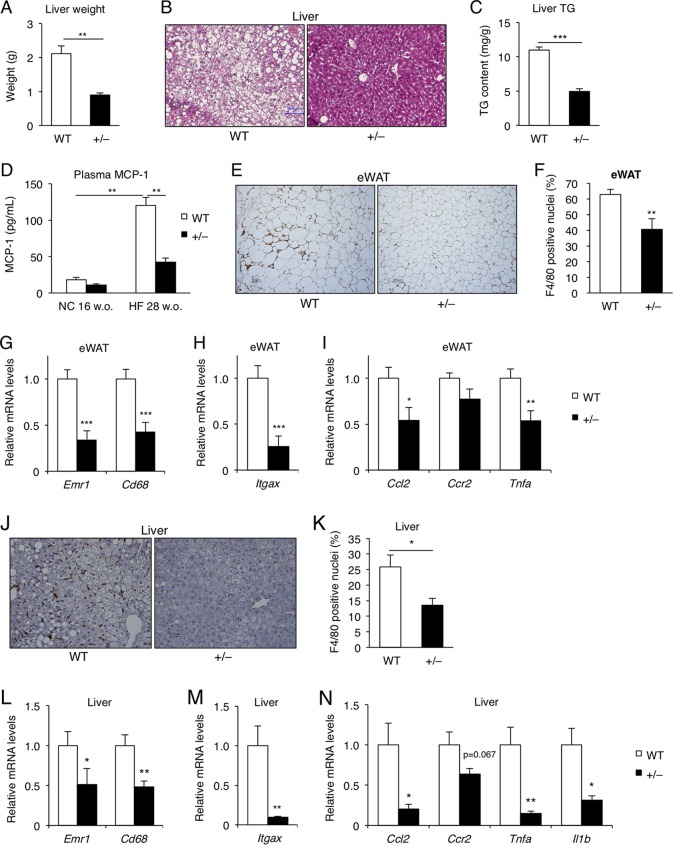
Hepatic steatosis and macrophage infiltration in both adipose tissue and liver were attenuated in *Wtap^+/^*^−^ mice. (A) Liver weight in 28-week-old mice on HFD for 20 weeks (*n* = 6). (B) H&E staining of liver from 28-week-old mice on HFD for 20 weeks. A representative section of each genotype is shown. Scale bar, 100 μm. (C) TG content of liver from 28-week-old mice on HFD for 20 weeks (*n* = 8). (D) Plasma MCP-1 levels in 28-week-old mice on HFD for 20 weeks (*n* = 6 to 8). (E and F) Immunohistochemistry with anti-F4/80 antibody in eWAT (E) and the ratios of anti-F4/80 antibody-positive nucleus counts (F) in 28-week-old mice on HFD for 20 weeks. Immunoreactivity for F4/80 is visualized as brown, whereas routine hematoxylin staining appears blue. Scale bars, 100 μm (*n* = 4 to 8). (G to I) The expression of macrophage-related and proinflammatory genes in the eWAT from 20-week-old mice on HFD for 12 weeks. Shown are *Emr1* (F4/80) and *Cd68* (G), an M1-like macrophage-related gene, *Itgax* (coding for CD11c) (H), and *Ccl2* (MCP-1), *Ccr2*, and *Tnf* (I) (*n* = 6 to 10). (J and K) Immunohistochemistry with anti-F4/80 antibody in liver (J) and the ratios of F4/80-positive nucleus counts (K) in 28-week-old mice on HFD for 20 weeks. Immunoreactivity for F4/80 is visualized as brown, whereas routine hematoxylin staining appears blue. Scale bars, 100 μm (*n* = 7 or 8). (L to N) The expression of macrophage-related and proinflammatory genes in liver from 20-week-old mice on HFD for 12 weeks. Shown are *Emr1* (F4/80) and *Cd68* (L), *Itgax* (M), and *Ccl2*, *Ccr2*, *Tnf*, and *Il1b* (N) (*n* = 8). Data represent means + SEM. *, *P* < 0.05; **, *P* < 0.01, ***, *P* < 0.001.

Steatosis was markedly improved in the liver of *Wtap*^+/−^ mice compared to that in WT mice, especially in those fed on HFD, as evaluated by liver weight (Fig. 12A), histology (Fig. 12B), and TG content (Fig. 12C).

Among the circulating adipokines that can affect inflammation and insulin sensitivity, HFD-induced elevation of circulating monocyte chemoattractant protein 1 (MCP-1) levels was dramatically suppressed in *Wtap*^+/−^ mice (Fig. 12D), while the adiponectin levels were not increased compared to those of WT mice (data not shown). Thus, we next analyzed macrophage infiltration in both adipose tissue and liver. Immunostaining with anti-F4/80 antibody (Fig. 12E and F) and expression analysis of the macrophage-related genes (Fig. 12G) in eWAT revealed decreased macrophage infiltration into the adipose tissue in *Wtap*^+/−^ mice compared to that of WT mice. This may be due to the decrease in M1-like macrophages, as shown by the decreased expression of *Itgax* (Fig. 12H). The lower expression levels of proinflammatory chemokines and cytokines such as MCP-1 and tumor necrosis factor alpha (TNF-α) also suggest the attenuation of inflammation in the WAT of *Wtap*^+/−^ mice (Fig. 12I). Similar to those in adipose tissue, the number of F4/80-positive cells (Fig. 12J and K) and the expression of macrophage-related genes (Fig. 12L) were decreased in the livers of *Wtap*^+/−^ mice. This may also be due to a decrease in M1-like macrophages (Fig. 12M); furthermore, expression of proinflammatory cytokines was also suppressed in the livers of *Wtap*^+/−^ mice (Fig. 12N). These data suggest that the inflammation induced by HFD in both liver and adipose tissue was ameliorated in *Wtap*^+/−^ mice compared to that in WT mice.

### *Wtap*^+/−^ mice exhibited better insulin sensitivity.

**FIG 13:**
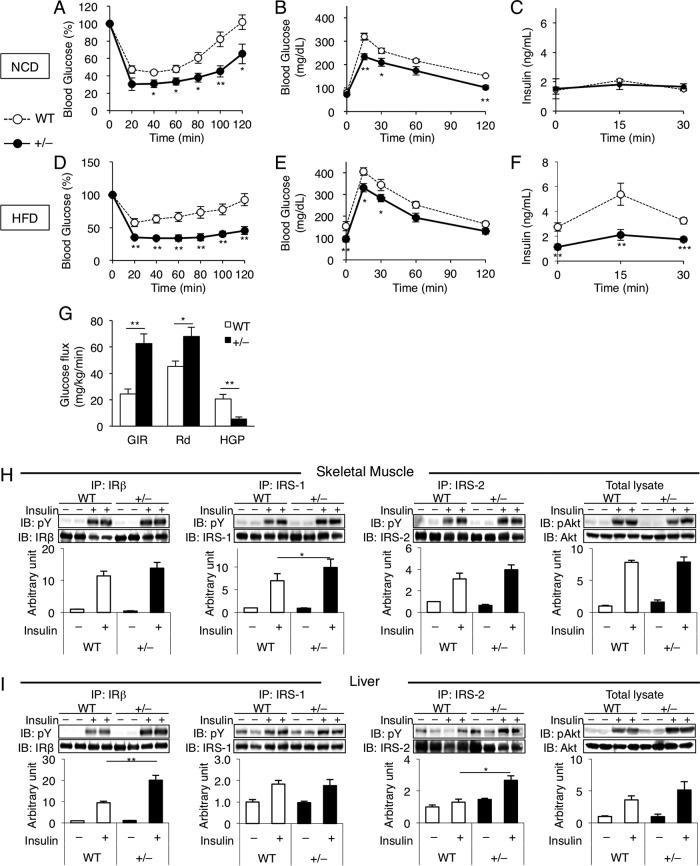
*Wtap^+/^*^−^ mice exhibited better glucose tolerance and insulin sensitivity. (A to C) ITT (A), OGTT (B), and plasma insulin from the OGTT (C) in 16-week-old mice fed on NCD (*n* = 7 to 14). (D to F) ITT (D), OGTT (E), and plasma insulin from the OGTT (F) in 16-week-old mice fed on HFD for 8 weeks (*n* = 4 to 9). (G) Hyperinsulinemic-euglycemic clamp study on 28- to 29-week-old mice fed on HFD for 20 to 21 weeks. GIR, glucose infusion rate; HGP, hepatic glucose production; Rd, rate of glucose disappearance (*n* = 5 or 6). (H and I) Insulin signaling pathway studies. Phosphorylation of insulin receptor β-subunit (IRβ), insulin receptor substrate (IRS-1 and IRS-2), and Akt induced by a bolus injection of insulin was assessed in skeletal muscles (H) and livers (I). IP, immunoprecipitation. Data represent means ± SEM (A to F) or means + SEM (G to I). *, *P* < 0.05; **, *P* < 0.01; ***, *P* < 0.001.

As a consequence of the attenuation of adiposity and inflammation, *Wtap*^+/−^ mice were more insulin sensitive than WT mice in insulin tolerance tests (ITT) (Fig. 13A and D) under both NCD (Fig. 13A to C) and HFD (Fig. 13D to F) feeding. Decreased plasma insulin levels (Fig. 13C and F) accompanied by the lower glucose levels (Fig. 13B and E) during the oral glucose tolerance test (OGTT) also suggest the improvement of insulin sensitivity in *Wtap*^+/−^ mice. Additionally, the hyperinsulinemic-euglycemic glucose clamp study revealed the improvement of insulin sensitivity in both the liver and skeletal muscle (Fig. 13G) of *Wtap*^+/−^ mice compared to that of WT mice. Furthermore, insulin signaling studies showed increased tyrosine phosphorylation of IRS-1 in skeletal muscle (Fig. 13H) and that of IRS-2 and the insulin receptor in the liver (Fig. 13I) of *Wtap*^+/−^ mice compared to WT mice.

### Reduced adiposity in parenchymal tissues contributes to improvement of tissue inflammation and insulin sensitivity in *Wtap*^+/−^ mice.

**FIG 14:**
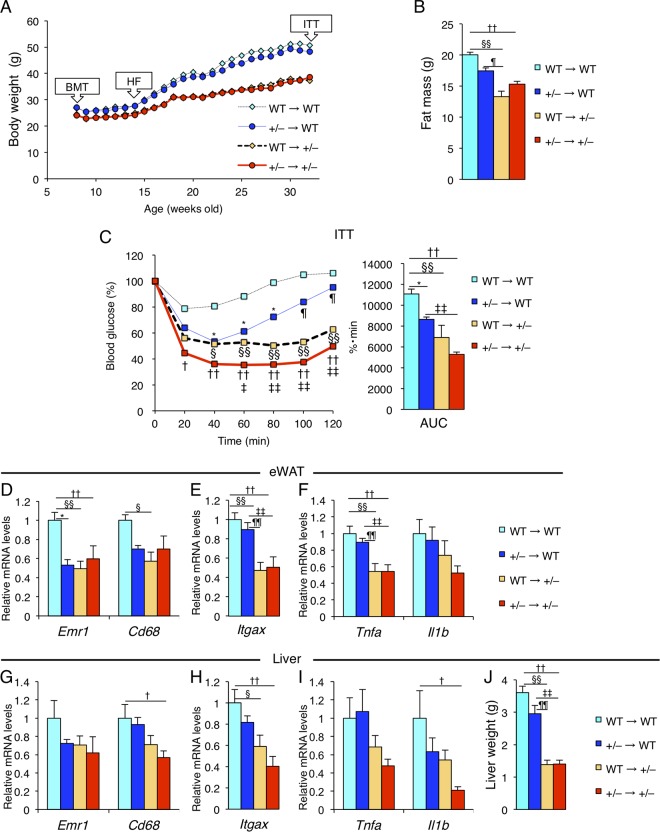
Reduced adiposity in parenchymal tissues contributes to improvement of tissue inflammation and insulin sensitivity in *Wtap*^+/−^ mice. Bone marrow transplantation (BMT) was performed on the indicated four groups, which represent BM from donor mice→recipient mice, in 34 week-old mice fed on HFD from 6 weeks after the transplantation for 20 weeks (*n* = 6 to 8). (A) Time course of the change in BW after BMT. (B) The fat mass as measured by DEXA. (C) The blood glucose levels in ITT (left) and its area under the curve (AUC) (right). (D to F) The gene expression of *Emr1* and *Cd68* (D), *Itgax* (E), and *Tnf* and *Il1b* (F) in eWAT. (G to I) The gene expression of *Emr1* and *Cd68* (G), *Itgax* (H), and *Tnf* and *Il1b* (I) in liver. (J) The weight of liver. Data represent means (A and C, left) and means + SEM (B; C, right; and D to J). *, *P* < 0.05 for WT→WT versus +/−→WT. *P* < 0.05 (§) and *P* < 0.01 (§§) for WT→WT versus WT→+/−. *P* < 0.05 (†) and *P* < 0.01 (††) for WT→WT versus +/−→+/−. *P* < 0.05 (¶) and *P* < 0.01 (¶¶) for +/−→WT versus WT→+/−. *P* < 0.05 (‡) and *P* < 0.01 (‡‡) for +/−→WT versus +/−→+/−.

The decrease in M1-like macrophages and improvement of inflammation in both adipose tissue and the liver of *Wtap*^+/−^ mice prompted us to address whether the changes of macrophages in adipose tissue and the liver were the primary effect of reduced WTAP in macrophages or the secondary effect of the suppression of obesity. To this end, we transplanted WT or *Wtap*^+/−^ bone marrow (BM) into both types of recipient mice and compared these four groups. *Wtap*^+/−^ recipient groups weighed much less (Fig. 14A), with much less fat (Fig. 14B), than WT recipient groups, while BM-specific heterozygous knockout of WTAP led to little or no change compared to the WT recipient mice with WT-derived BM. The ITT of each group showed the results in relation to their BW change; large improvements of insulin sensitivity were observed in the *Wtap*^+/−^ recipient groups, while the beneficial effects of *Wtap*^+/−^ BM in the WT recipient mice were limited (Fig. 14C). Furthermore, gene expression analyses showed the macrophage markers and their related inflammatory genes were suppressed in both adipose tissue (Fig. 14D to F) and the liver (Fig. 14G to I), and hepatic steatosis (Fig. 14J) was improved in the *Wtap*^+/−^ recipient groups almost to the same extent as that observed in *Wtap*^+/−^ mice. These data suggest that haploinsufficiency of WTAP in parenchymal tissues has a stronger impact on the improvement of tissue inflammation and insulin sensitivity associated with protection against BW gain than WTAP haploinsufficiency in BM-derived cells, including macrophages.

## DISCUSSION

Understanding the molecular mechanisms regulating adipocyte differentiation and maturation is a key step to developing useful therapeutic strategies for the treatment of obesity and its associated diseases, such as T2D. Although thiazolidinediones, which are agonists for PPARγ, a master regulator of adipocyte differentiation and maturation, were developed and widely used for the treatment of T2D, this class of drugs often causes side effects, such as weight gain, bone fracture, and fluid retention. Therefore, identification of another molecular target, the modulation of which leads to protection from obesity, is desirable.

In this report, we have shown the function of WTAP as an important factor for adipocyte differentiation and maturation. WTAP, together with METTL3 and METTL14, coordinates the cell cycle, promoting MCE in adipocyte differentiation. WTAP reduction can lead to amelioration of HFD-induced obesity *in vivo* with suppression of the size and number of adipocytes, resulting in better insulin sensitivity than that of WT mice. Thus, the WMM complex plays an essential role in regulating adiposity *in vivo*, and modulation of its expression and function can be a novel therapeutic strategy for the treatment of obesity and its associated diseases.

However, *in vivo* studies have a limitation in that WTAP is ubiquitously expressed and systemic heterozygous knockout mice were used. Therefore, there is a possibility that the reduction of WTAP affects tissues other than adipocytes, and it is difficult to distinguish which organ is primarily affected. For example, in the bone marrow transplantation (BMT) study, we have shown that the effects of WTAP reduction in BM-derived cells, including macrophages, was limited compared to those in parenchymal tissues (Fig. 14), but we could not distinguish the effect of WTAP reduction within parenchymal tissues, especially between adipose tissue and liver. On the other hand, increased energy expenditure with upregulation of UCP-1 protein and its related instances of gene expression in BAT could also contribute to the protection from DIO in *Wtap*^+/−^ mice (Fig. 11B to E). Although some brown fat-related gene expression levels were elevated in scWAT of *Wtap*^+/−^ mice, their UCP-1 protein levels were far less than those in BAT and were almost undetectable by Western blotting and immunohistochemistry in scWAT of both WT and *Wtap*^+/−^ mice (Fig. 11F to H). Thus, the elevated function of BAT is likely to contribute mainly to the increased energy expenditure, but it remains unknown whether reduction of WTAP directly affects BAT. Nevertheless, taking *in vitro* data and *in vivo* data together (Fig. 1, 4, and 10), it can be suggested that smaller size and number of adipocytes in *Wtap*^+/−^ mice are, at least in part, a direct effect of WTAP reduction on preadipocytes, which could have a significant contribution to the improvement of insulin sensitivity and inflammation in *Wtap*^+/−^ mice.

Regarding the molecular mechanisms by which WTAP regulates adipocyte differentiation, our data suggest that WTAP cooperates with METTL3 and METTL14 to promote cell cycle transition during MCE. Knockdown of WTAP, METTL3, METTL14, or cyclin A2 in 3T3-L1 cells and MEF-*Wtap*^+/−^ similarly leads to cell cycle arrest in MCE and impaired adipocyte differentiation *in vitro*. Cyclin A2 is upregulated specifically during MCE in adipocyte differentiation in cultured adipocyte cell lines or MEF, and *Ccna2* expression is upregulated in SVCs from the WAT of WT mice after 2 weeks on HFD, whereas it is suppressed in SVCs from the WAT of *Wtap*^+/−^ mice. These data indicate that cyclin A2 is required for the regulation of the MCE in adipocyte differentiation *in vitro. In vivo* data may also suggest an involvement of cyclin A2 in expansion of fat mass. On the other hand, WTAP reduction leads to impaired *Ccna2* upregulation and cell cycle arrest in the MCE of adipocyte differentiation *in vitro* and *in vivo*. Since METTL3 and METTL14 are increased and distributed in the nucleus by the induction of adipocyte differentiation in a WTAP-dependent fashion and the nuclear increase of WTAP is dependent on RNA, it is suggested that WTAP recruits METTL3 and METTL14 to mRNA, thereby regulating expression of genes involved in adipocyte differentiation. Furthermore, knockdown of METTL3 and METTL14 in 3T3-L1 cells, even with upregulated WTAP, leads to suppressed *Ccna2* expression and cell cycle arrest in MCE, resulting in impaired adipocyte differentiation. Indeed, cell cycle regulation has been suggested to be one of the m^6^A-RNA-related molecular functions by many previous reports (7–9, 31–36). Thus, a reduction of WTAP may impair the recruitment of METTL3 and METTL14 to mRNA, leading to dysregulation of the production of their downstream cell cycle-related molecules, including cyclin A2.

As observed in inhibition of other cell cycle-regulating molecules (21–24, 37, 38), the impairment of cell cycle transition by suppression of WTAP leads to downregulation of *Pparg* and its related gene expression *in vivo* and *in vitro*. Indeed, there are considerable similarities in the phenotypes between *Wtap*^+/−^ and *Pparg*^+/−^ mice (39), including smaller adipocytes, decreased liver weight, and less food intake (per individual mouse) with better insulin sensitivity.

However, it still needs to be elucidated whether cyclin A2 is directory regulated by the WMM complex in adipocyte differentiation by installation of m^6^A on *Ccna2* mRNA. Since in a previous comprehensive study with photoactivatable ribonucleoside-enhanced cross-linking and immunoprecipitation (PAR-CLIP) in HeLa cells *Ccna2* was not significantly enriched as a candidate target of WMM while considerable numbers of cyclins and CDKs were enriched (8), it is possible that regulation of *Ccna2* expression by WMM is mediated by m^6^A-mRNA methylation of other cell cycle-related molecules.

The results of the function of methyltransferase complex WMM in adipocyte differentiation shown in this study seem to be contrary to some studies of FTO, which has been shown to be a demethylase of m^6^A (12) and promotes adipogenesis *in vitro* and *in vivo* (13–15). However, it is possible that different genes for adipocyte differentiation are targeted and regulated by m^6^A-methyltransferase WMM complex and demethylase FTO, respectively. Indeed, it has also been reported that expression of many genes is regulated by either METTL3 or FTO alone rather than coregulated by both in adipocyte differentiation of 3T3-L1 cells (8). Thus, the regulation of m^6^A-RNA methylation by the WMM complex as well as FTO may be involved in pathogenesis of human obesity or T2D, although further studies are needed to identify the main target of m^6^A-RNA methylation by the WMM complex and to elucidate how different genes are regulated by both m^6^A-RNA methylation and demethylation to understand the mechanism of subsequent cell cycle regulation and adipocyte differentiation and maturation. Further, whether other tissues, such as BAT, macrophage, or hepatocytes, are directly affected by the WMM complex should also be investigated.

The biological functions of nuclear speckles have been considered to be acting as a storage site of splicing factors and to play a role in supplying these factors to nucleoplasm, active splicing, or posttranscriptional RNA modification sites (40), and the intracellular localization of WTAP in not only nuclear speckle but also nucleoplasm has been previously described (16). In our data, both the induction of adipocyte differentiation with DMI and the knockdown of WMM affected their immunofluorescence staining in not only nuclear speckles but also nucleoplasm (Fig. 6B and 8C), and despite remaining low levels of METTL3 or METTL14 proteins in nuclear speckles against their siRNAs (Fig. 8B and C), the effects of these knockdowns biologically worked well enough (Fig. 9D to G). Therefore, these data suggest a role of active posttranscriptional regulation of the WMM complex by being distributed in nucleoplasm, similar to other nuclear speckle proteins, which responded to the induction of adipocyte differentiation.

It has been demonstrated that METTL3 and METTL14 form a stable heterodimer whose methyltransferase activity is much higher than that of individual protein (8), and recent studies also have shed light on its details structurally and functionally *in vitro* (26, 41, 42). In our study, knockdown of METTL3 or METTL14 impaired adipocyte differentiation, and the double knockdown showed additive effects (Fig. 9D to G). Moreover, the single knockdown of METTL3 or METTL14 impairs the other's protein level (Fig. 8B and 9C), and the single overexpression of METTL3 or METTL14 showed much less efficacy than cooverexpression of both proteins (Fig. 9H). Thus, our data also suggest the functional importance of heterodimer formation of METTL3 and METTL14 in adipocyte differentiation, which is consistent with recent reports (26, 41, 42).

Although many more investigations are needed, our data collectively suggest that intervention of WTAP, METTL3, METTL14, or an undetermined target of cell cycle-related molecules is a strategy for preventing and treating obesity or obesity-related diseases.

## MATERIALS AND METHODS

### Cells and cell culture.

3T3-L1, 3T3-F442A, COS cells, and MEF were maintained in Dulbecco's modified Eagle's medium (DMEM) (Sigma) supplemented with 10% fetal bovine serum (FBS) (Biowest). For adipocyte differentiation of 3T3-L1 or MEF, the cells at confluence were treated with dexamethasone (1 μM), IBMX (0.5 mM), and insulin (5 μg/ml) for 48 h, followed by treatment with insulin alone. For 3T3-F442A differentiation, the cells were treated with 5 μg/ml of insulin.

### Quantitative real-time PCR.

The total RNA was prepared using the RNeasy kit (Qiagen). cDNA was prepared using high-capacity cDNA reverse transcription kits (Applied Biosystems). Quantitative real-time PCR was performed with an ABI Prism 7900HT using TaqMan gene expression assays with PCR master mix reagent (Applied Biosystems), except for the quantification of *Wtap*, *Ccna2*, *Prdm16*, and *Nfia* mRNA, which was performed with the Power SYBR green PCR master mix (Applied Biosystems). The sequences of the primers are the following: *Wtap*-fwd, ACGCAGGGAGAACATTCTTG; *Wtap*-rev, CACACTCGGCTGCTGAACT; *Ccna2*-fwd, CTTGGCTGCACCAACAGTAA; *Ccna2*-rev, ATGACTCAGGCCAGCTCTGT; *Prdm16*-fwd, TGAAGGAGGCCGACTTTG; *Prdm16*-rev, TCTCCTGGGATGACACCTCT; *Nfia*-fwd, CCATTTTACACAGGCCAAGG; *Nfia*-rev, TGGCTGGGTGTGAGAAGTAAG. mRNA levels were normalized to those of β-actin (*Actb*) (43) or cyclophilin A (*Ppia*) (44).

### Protein knockdown with siRNA and adeno-shRNA *in vitro*.

siRNA was added to 3T3-L1 cells as described by Tong et al. (45), whose protocol was also applied for the adenovirus-mediated WTAP knockdown. Briefly, siRNA oligonucleotides with Lipofectamine RNAiMAX (Invitrogen) or adeno-short hairpin RNA (shRNA) were administered to 3T3-L1 cells at about 50% confluence 2 days before the induction of differentiation with DMI, and then the cells were trypsinized and plated at the cell density generating confluent monolayers at 24 h before the induction. The cells were harvested and analyzed at each indicated time point. Silencer siRNA, Silencer select siRNA, and its control siRNA oligonucleotides (Applied Biosystems) were used for *Ccna2* knockdown at standard concentrations. For the knockdown of *Mettl3*, *Mettl14*, and *Wtap*, Silencer select siRNA and its control siRNA oligonucleotides were used at 40 nM. To compare the double knockdown of *Mettl3* and *Mettl14* with their respective single knockdowns, the same concentration of the control siRNA was added for the single knockdown.

### Adenovirus and plasmids.

The shRNA construct of mouse WTAP was targeted at the sequence AAGCTTTGGAGGGAAAGTACA, which was inserted into a pENTR4PURhU6icas vector (pENTR4–WTAP-shRNA). Its hU6 promoter and shRNA construct were transferred into pAxcwit, generating adeno-shWTAP, according to the manufacturer's instructions (TaKaRa Bio). An adenovirus-encoding shRNA sequence for green fluorescent protein (GFP) was used for its control (44). The cDNA of mouse WTAP was subcloned and inserted into pGEX-4T1 (GE Healthcare Life Sciences). The cloning primers of WTAP were the following: fwd, ATGACCAACGAAGAACCTCT; rev, TTACAAAACTGCACCCTGTACA. The deletion mutants of GST-WTAP were generated by the KOD plus mutagenesis kit (Toyobo). The pEZ-FLAG-mMETTL3 vector was purchased from GeneCopoeia. pEZ-FLAG-mMETTL14 was generated by substitution of subcloned mouse METTL14.

### Protein extraction, RNase treatment, and immunoblotting.

For *in vitro* examination, cells were harvested and homogenized with buffer containing 25 mM Tris-HCl (pH 7.4), 10 mM Na_3_VO_4_, 100 mM NaF, 10 mM Na_4_P_2_O_7_, 10 mM EGTA, 10 mM EDTA, protease inhibitor cocktail (Roche). For animal tissues, the buffer was modified to 50 mM HEPES (pH 7.4), 2 mM Na_3_VO_4_, 10 mM NaF, 10 mM Na_4_P_2_O_7_, 2 mM EGTA, 2 mM EDTA. Total cell lysates, including nuclear protein, were extracted with 420 mM NaCl and 1% NP-40. For crude fractionation of cytosolic/nuclear protein and RNase treatment (Fig. 8A), the cells were harvested with a hypotonic buffer containing 100 mM HEPES, 15 mM MgCl_2_, 100 mM KCl and incubated on ice for 15 min, and then 0.06% NP-40 was added. The lysates were treated with 1 mg/ml RNase A (Sigma-Aldrich) and then equally divided into two parts; one was used as the total fraction, and the other was used to extract cytosolic and nuclear protein. The supernatant of the latter, after spin-down at 450 × *g* for 5 min, was used as the cytosolic fraction, and the pellet was resolved with the same volume of the hypotonic buffer as that for the nuclear fraction. NaCl and NP-40 were added to every fraction at 420 mM and 1% final concentration, respectively. For the protein fractionation shown in Fig. 7B and C and 8B, the ProteoExtract subcellular proteome extraction kit was used according to the manufacturer's instructions (Merck). After incubation and centrifugation, the supernatants were collected. The protein samples were prepared with Laemmli sample buffer, separated on polyacrylamide gels, and transferred to polyvinylidene difluoride (PVDF) membrane. Membranes were probed with anti-cyclin A (Santa Cruz Biotechnology), anti-METTL3 (Abnova), anti-METTL14 (Sigma), anti-UCP-1 (Abcam), and anti-WTAP antibody. The rabbit anti-WTAP antibody for immunoblotting (IB) was a kind gift from K. Horiuchi (17). Anti-lamin A/C (Cell Signaling Technology) antibody was used as a loading control of nuclear protein, and anti-tubulin-α (Thermo Fisher Scientific) or anti-glyceraldehyde-3-phosphate dehydrogenase (anti-GAPDH) (Cell Signaling Technology) antibody was used as that of cytosolic protein. The blot was detected using a chemiluminescence system (GE Healthcare Life Sciences) and quantified by densitometry using ImageJ software.

### Cell cycle analysis.

For cell cycle analysis of 3T3-L1 cells, 40 h after the induction of adipocyte differentiation with DMI, the cells were harvested with trypsin-EDTA and washed with phosphate-buffered saline (PBS). After fixation with cold 75% ethanol, the cells were washed with PBS and suspended with the staining buffer, which contained 100 mM Tris (pH 7.4), 150 mM NaCl, 1 mM CaCl_2_, 0.5 mM MgCl_2_, and 0.1% NP-40 with 0.5% RNase A. For cell cycle analysis of SVCs, since just-isolated cells or primary SVCs were inappropriate for flow cytometric analysis of the cell cycle, isolated SVCs from scWAT of *Wtap*^+/−^ and WT mice were cultured. At the second passage they were plated at confluent density, and on the following day the cells were stimulated with standard DMI. Twenty hours later the cells were harvested and stained with anti-CD31–eFluor 450 (eBioscience) and the APC mouse lineage antibody cocktail (BD Biosciences). After fixation by 4% paraformaldehyde phosphate buffer, the cells were incubated with 0.1% NP-40 and RNase A. The cells stained with propidium iodide (Invitrogen) were analyzed on a FACSCalibur or FACSVerse flow cytometer (Becton Dickinson). For SVCs the cell cycle was analyzed for the lineage- and CD31-double negative population. The percentage distribution of each cell cycle period was determined using FlowJo software.

### Oil Red O staining.

Maturated 3T3-L1 cells at 10 days after DMI induction were stained with Oil Red O as previously described (46).

### Embryonic fibroblasts.

Thirteen to 16 days postcoitus, embryos were cut into small pieces with scissors and digested by 0.25% trypsin–EDTA at 37°C with stirring for 30 min. The cells were cultured in DMEM (Sigma) with 10% FBS and stored in liquid nitrogen. Cells showing good growth with a low number of passages were used in the experiments.

### Pulldown assay.

GST-fused WTAP, and its deletion mutant proteins as bait, were produced by SoluBL21-competent Escherichia coli (Genlantis). pEZ-FLAG-mMETTL3 or pEZ-FLAG-mMETTL14 was transfected into COS cells, and their lysates (prey) were incubated with GST-WTAP or its mutant proteins immobilized on glutathione-Sepharose 4B (GE Healthcare Life Sciences). After extensive washing, the protein complex was eluted with 10 mg/ml reduced glutathione and assessed by Western blotting with anti-METTL3 (Abnova), METTL14, and FLAG M2 antibody (Sigma). NP-40 (0.02%) was used for the lysis buffer, the binding buffer, and the washing buffer in this assay.

### Immunofluorescence staining.

3T3-L1 cells were grown on 0.2% gelatin-coated coverslips and induced to differentiate into adipocytes with standard DMI. At the indicated time point cells were fixed with 95% ethanol, followed by permeabilization with 0.1% Triton X-100 and 0.5% NP-40 on ice. After blocking with 5% nonfat dried milk in TBST, the coverslips were incubated with primary antibody, namely, mouse anti-METTL3 (Abnova), rabbit anti-METTL14 (Sigma), goat anti-WTAP (Santa Cruz), mouse-anti-SC-35 (Abcam), or mouse anti-Smith antigen (Thermo Fisher Scientific), overnight at 4°C. The coverslips then were incubated with fluorescent dye-conjugated secondary antibody, namely, anti-mouse IgG–Alexa Fluor 488 (Cell Signaling Technology), anti-rabbit IgG–Alexa Fluor 594, or anti-goat IgG–Alexa Fluor 647 (Abcam), for 0.5 h at 37°C and mounted with DAPI-containing medium (Life Technologies). Fluorescent images were acquired using an FV10i confocal microscope (Olympus).

### Animals.

Mice were housed under a 12-h light-dark cycle and given *ad libitum* access to NCD (MF; Oriental Yeast) consisting of 25% (wt/wt) protein, 53% carbohydrate, 6% fat, and 8% water, or an HFD (HF-32; CLEA Japan) consisting of 25.5% (wt/wt) protein, 2.9% fiber, 4.0% ash, 29.4% carbohydrate, 32% fat, and 6.2% water. *db/db* mice and their control *m/m* mice were purchased from CLEA Japan. *Wtap* knockout mice were described previously (17) and were maintained on the C57BL/6 background. Because *Wtap* homozygous null (*Wtap*^−*/*−^) mice are embryonically lethal (17, 29), *Wtap* heterozygous knockout (*Wtap^+/^*^−^) mice were used for all our experiments, and their littermates (*Wtap^+/+^*) were used as the WT control. All experiments in this study were performed on male mice, except for the female mice used in the BMT study as BM donors. The animal care and experimental procedures were approved by the Animal Care Committee of the University of Tokyo.

### Body composition.

The body compositions (lean mass and fat mass) of mice were determined by DEXA with a LUNAR PixiMus2 scanner (GE Healthcare Life Sciences).

### Adipocyte size and number.

The cranial distal part of eWAT (30 to 50 mg), after removing just the tip portion, was fixed with osmium tetroxide for 3 days at 37°C. As previously described (47), fixed and dispersed cells were passed through a 250-μm nylon filter to remove fibrous elements, followed by a 25-μm nylon filter to trap fixed adipocytes (with a diameter of 25 to 250 μm); the cells were then washed with PBS. The cell suspensions were analyzed for 2 min using a Coulter counter (Multisizer 3; Beckman Coulter) with a 400-μm aperture tube to measure the volume and diameter of the individual cells. The total adipocyte number in eWAT was calculated by measuring the total WAT weight, mean adipocyte volume, and adipocyte density (0.948 mg/ml) as described previously (20).

### Food intake and oxygen consumption.

For monitoring food intake, the food of mice fed on HFD in individual cages was weighed once daily for seven consecutive days. Oxygen consumption was measured every 3 min for 24 h in the fasting mice using an O_2_/CO_2_ metabolism measurement device (model MK-5000; Muromachi Kikai). After being fed on HFD for 1 to 2 weeks, each mouse was placed in a sealed chamber (560-ml volume) with an airflow rate of 500 ml/min at room temperature, and cumulative recordings were collected over 36 h. The amount of oxygen consumed was converted to milliliters per minute by multiplying it by the flow rate.

### Fractionation of adipocytes and SVCs.

For gene expression analysis, adipocytes and SVCs were fractionated from eWAT as previously described (1). For cell cycle analysis, SVCs were isolated from scWAT of *Wtap*^+/−^ and WT mice fed on HFD using an adipose tissue dissociation kit and a gentleMACS dissociator according to the manufacturer's instructions (Miltenyi Biotec).

### Blood sample assays.

Plasma MCP-1 and leptin concentrations were quantified by enzyme-linked immunosorbent assay (ELISA) (R&D Systems). The plasma T-Chol, TG, and FFA were assayed by enzymatic methods (Wako Pure Chemical Industries). Blood samples were obtained after 24 h of fasting for plasma MCP-1 and leptin analysis and under *ad libitum* feeding conditions for plasma T-Chol, TG, and FFA analysis.

### Insulin signaling study *in vivo*.

Mice were anesthetized after 24 h of fasting, and 5 U of human insulin (Humalin R; Eli Lilly) was injected into the inferior vena cava. After 5 min, the liver and hind limb muscles were dissected and immediately frozen in liquid nitrogen. The extracted proteins were immunoprecipitated by anti-IRβ (Santa Cruz Biotechnology), IRS-1, and IRS-2 (Millipore) and immunoblotted by anti-IRS-1, IRS-2, and phosphotyrosine (4G10) (Millipore). For phospho-Akt (Ser-473) and Akt analysis, the extracted total proteins were immunoblotted with their respective antibodies (Cell Signaling Technology).

### Histological analysis.

For immunohistochemistry of F4/80, eWAT samples were fixed with 4% paraformaldehyde in PBS overnight. Adipose tissues were embedded in paraffin and cut into 5-μm sections at 50-μm intervals. After deparaffinization, the sections were stained with rat monoclonal anti-F4/80 antibody (Serotec), followed by incubation with the Vectastain Elite ABC kit, visualization with the ImmPACT DAB substrate kit (Vector Laboratories), and counterstaining with hematoxylin and eosin (H&E). Livers were cut into 4-μm frozen sections at 50-μm intervals. For the observation of steatosis, liver sections were stained with H&E, and for macrophage analysis, they were stained with F4/80 antibody followed by counterstaining with hematoxylin. The total number of nuclei and the number of F4/80-positive nuclei were counted in four different high-power fields from each of four different sections. The nuclei of more than 1,000 cells in eWAT and more than 2,000 cells in the liver per mouse were counted. Immunohistochemistry of BAT and scWAT with anti-UCP-1 antibody (Abcam) was performed by the Nara Pathology Research Institute Co., Ltd.

### Measurement of triglyceride contents of the liver.

Hepatic TG contents were determined as described previously (48).

### Metabolic studies.

For OGTT, mice were fasted for 16 h and loaded with oral glucose at 1.0 mg/g BW. ITT was performed by intraperitoneal injection of 0.825 mU/g BW of human insulin (Humalin R; Eli Lilly) for mice fed on NCD and 1.4375 mU/g BW for mice fed on HFD. Blood samples were taken at different time points, and the glucose concentrations were measured at the indicated time points with an automatic glucometer (Sanwa Kagaku Kenkyusho). Plasma insulin levels were determined using an insulin radioimmunoassay kit (Institute of Isotopes).

### Hyperinsulinemic-euglycemic clamp study.

As described previously (49), 3 to 4 days before the study, an infusion catheter was inserted into the right jugular vein under general anesthesia with sodium pentobarbital. The studies were performed on mice under conscious and unstressed conditions after a 6-h fast. After 120 min of basal glucose turnover study (data not shown) with 0.05 μCi/min [3-^3^H]glucose (PerkinElmer) and following a bolus injection of 10 μCi of [3-^3^H]glucose, the clamp study was applied for 120 min with a continuous infusion of insulin (Humalin R; Eli Lilly) at a rate of 15 mU/kg/min and [3-^3^H]glucose at a rate of 0.1 μCi/min. The plasma glucose concentration was monitored every 5 min and maintained at 100 to 120 mg/dl by infusion of 50% glucose at a variable rate. Blood samples from tail-tip bleeds were collected 80, 90, 100, 110, and 120 min after the onset of the clamp, and measurements of [3-^3^H]glucose-specific activity and calculation of the rates of glucose disappearance (Rd) and hepatic glucose production (HGP) rates were performed as described previously (50).

### Bone marrow transplantation.

BM cells were collected from the femurs and tibiae of the female mice at 8 weeks of age. The nucleated cells were counted and injected intravenously (5 × 10^6^ cells/recipient) into lethally irradiated (10 Gy) male recipient mice at 8 weeks of age. Six weeks after transplantation, the mice were fed on HFD for 20 weeks before metabolic analysis. We confirmed that >95% of the leukocytes in the peripheral blood were reconstituted by donor cells (data not shown) following a previously described chimerism assay (51).

### Statistics.

Data were analyzed by two-tailed Student's *t* test for comparison between two groups or analysis of variance (ANOVA), followed by Tukey-Kramer's *post hoc* test with Statcel2 software for comparison of multiple groups. A *P* value of less than 0.05 was considered statistically significant. Error bars represent the standard errors of the means (SEM).
